# The Origin of a New Sex Chromosome by Introgression between Two Stickleback Fishes

**DOI:** 10.1093/molbev/msy181

**Published:** 2018-10-01

**Authors:** Groves Dixon, Jun Kitano, Mark Kirkpatrick

**Affiliations:** 1 Department of Integrative Biology, University of Texas, Austin, TX; 2 Division of Ecological Genetics, Department of Population Genetics, National Institute of Genetics, Mishima, Shizuoka, Japan

**Keywords:** sex chromosome, introgression, stickleback, degeneration, sex-biased expression

## Abstract

Introgression is increasingly recognized as a source of genetic diversity that fuels adaptation. Its role in the evolution of sex chromosomes, however, is not well known. Here, we confirm the hypothesis that the Y chromosome in the ninespine stickleback, *Pungitius pungitius*, was established by introgression from the Amur stickleback, *P. sinensis*. Using whole genome resequencing, we identified a large region of Chr 12 in *P. pungitius* that is diverged between males and females. Within but not outside of this region, several lines of evidence show that the Y chromosome of *P. pungitius* shares a most recent common ancestor not with the X chromosome, but with the homologous chromosome in *P. sinensis*. Accumulation of repetitive elements and gene expression changes on the new Y are consistent with a young sex chromosome in early stages of degeneration, but other hallmarks of Y chromosomes have not yet appeared. Our findings indicate that porous species boundaries can trigger rapid sex chromosome evolution.

## Introduction

New sex chromosomes are typically thought to originate by mutation, for instance with the transposition of a sex determining gene to an autosome (van Doorn and Kirkpatrick 2007; Vicoso and Bachtrog 2013). Another possible source of new sex chromosomes is introgression. But sex chromosomes are generally thought to be unlikely to introgress between species because they frequently carry genetic incompatibilities (Coyne and Orr 2004; Neafsey et al. 2015; Muirhead and Presgraves 2016; Payseur and Rieseberg 2016).

Several studies, however, suggest that sex chromosomes do occasionally introgress. A sex determining region of cottonwoods shows evidence of moving between *Populus alba* and *P. tremula* (Stölting et al. 2013). Y-chromosome introgression has also been reported between primates (Tosi et al. 2002). Although they are not sex chromosomes in the strictest sense, degenerate mating-type chromosomes in yeast, *Neurospora tetrasperma*, have been replaced repeatedly via introgression of homologous copies from closely related species (Corcoran et al. 2016).

In addition to the examples above, Natri (2015) hypothesized that the Y-chromosome of the ninespine stickleback, *Pungitius pungitius*, originated from the closely related *Pungitius sinensis*. Using 56 microsatellite loci, she showed that *P. pungitius* males are heterogametic, with genetically diverged X and Y chromosomes. Linkage mapping revealed that a large inversion (15 Mb of the 21-Mb length chromosome) blocks recombination between the X and Y. Comparison with the karyotype of the threespine stickleback (*Gasterostreus aculeatus*) showed that the arrangement on the *P. pungitius* Y is ancestral, while the X carries an inversion that fixed at some point after divergence from *G. aculeatus*. Based on several analyses using the microsatellites, she concluded that the Y chromosome in *P. pungitius* is derived from *P. sinensis*. This situation is all the more intriguing because *P. sinensis* is thought to have a ZW sex determination system (Natri 2015). The ZW locus was mapped to a section of chromosome Chr 7 that is fused to Chr 12 in the genus *Pungitius* (Natri 2015).

The potential for such an introgression event is supported by reports that *P. pungitius* and *P. sinensis* can indeed hybridize, both in the wild (Ishikawa et al. 2013) and the laboratory (Takahashi et al. 2005; Meguro et al. 2016). Although hybrid males are sterile, females are fertile (Takahashi et al. 2005).

This remarkable situation immediately raises questions. Does whole genome sequencing confirm the inferences from microsatellites about the origin of the Y? How do recent changes in our understanding of the phylogenetic relations between *P. pungitius* and *P. sinensis* alter conclusions about the introgression? Is there evidence for introgression elsewhere in the genome? Did the introgressing chromosome act as a Y from the very start, or did it evolve into a sex chromosome later? Does the new Y chromosome show signs of the degeneration often seen on Y chromosomes (Bachtrog 2013)?

These questions motivated us to revisit the origin of the Y chromosome in *P. pungitius*. We collected whole genome sequences from 9 to 15 individuals of each sex of *P. pungitius* and *P. sinensis*, as well as the closely related Sakhalin stickleback, *P. tymensis*. For simplicity, we will refer to these by their specific names: *pungitius, sinensis*, and *tymensis*. First, we confirm that the large segment of Chromosome 12 that corresponds to the putative inversion is indeed the sex determining region in *pungitius*. This region is unique in the genome: it is highly diverged between males and females, and has gene trees whose topologies are characteristic of X and Y chromosomes. Second, we find evidence of gene flow between *pungitius* and *sinensis*. Third, the introgression hypothesis is confirmed. *P. pungitius* males show high levels of shared ancestry with *sinensis* within the inverted region but not elsewhere, and inferred Y-chromosome haplotypes share a most recent common ancestor with *sinensis*. Finally, examination of the new Y chromosome indicates it is in a very early stage of degeneration, and has evolved regulatory differences from the X that contribute to sex-specific gene expression.

## Results

### Chromosome 12 is a Sex Chromosome in *P. pungitius*

We obtained whole genome sequences from 30 *pungitius* (15 females and 15 males), 23 *tymensis* (10 females and 13 males), and 20 *sinensis* (9 females and 11 males). Reads were mapped to the reference genome of the threespine stickleback (*Gasterosteus aculeatus*) (Glazer et al. 2015). (The chromosome numbering used throughout the manuscript refers this reference genome.) The sequences were then phased bioinformatically (see Materials and Methods) (Browning and Browning 2007).

**Fig. 1.**
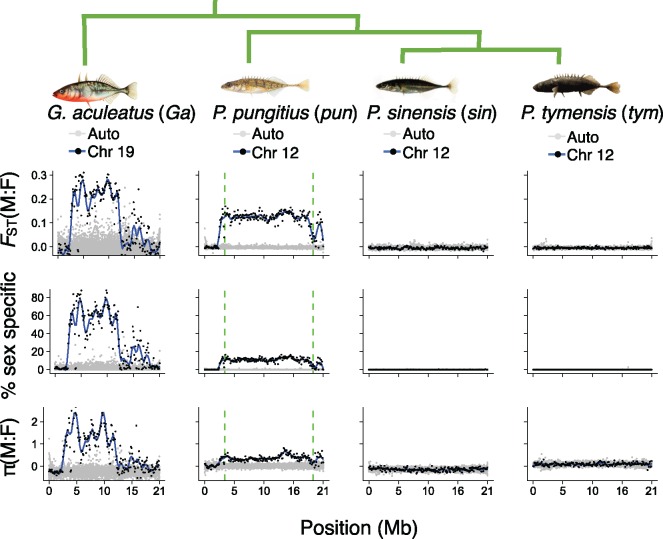
Evidence of a large nonrecombining region on Chr 12 in *Pungitius pungitius*. Points represent 100-kb windows. Data from confirmed or hypothesized sex chromosomes are shown as black points with a blue loess regression; data from all other chromosomes are in gray. Results for Chr 12 are shown for the three *Pungitius* species. Chr 19 for *Gasterostreus aculeatus*, which has a well-established SDR with known strata, is shown as a control. The three statistics are: F_ST_ between males and females, the percentage of SNPs with a minor allele frequency >0.1 in one sex and absent in the other, and the log_2_ of the male:female ratio for nucleotide diversity (π). The vertical dashed lines for *P. pungitius* show the inversion breakpoints at 3.5 and 18.9 Mb identified by Natri (2015). Chromosome positions refer to the sex chromosome, and autosomes have been rescaled to the same length. Abbreviations following the latin binomials are used in later figures.

Comparison of genotypes between males and females revealed a region on Chr 12 with clear elevation of *F*_ST_ in *pungitius* but not in the other two species (fig. 1). The region with extensive divergence in *pungitius* corresponds to the 15-Mb inversion described by Natri (2015). We therefore confirm that this is the sex determining region, or SDR, within which the X and Y do not recombine. To the left of the inversion, males and females are not diverged, so we infer this is a recombining pseudoautosomal region, or PAR. To the right of the inversion, there is evidence of reduced, but not entirely suppressed recombination: *F*_ST_ and sex-specific alleles are inflated relative to the PAR and autosomes, but lower than in the SDR. Similar results were observed for SNPs called from two publicly available RNA-seq data sets: one from *pungitius* sampled from the same population as this study (White et al. 2015), and another from *pungitius* sampled from St. Lawrence Island, Alaska (von Hippel et al. 2018) (supplementary fig. S1, Supplementary Material online). In contrast, we did not find any evidence of an SDR in *sinensis* or *tymensis*, either on Chr 12 or elsewhere in their genomes (fig. 1).

Phylogenies constructed from each autosome showed that *sinensis* and *tymensis* are sister species, and *pungitius* is an outgroup (fig. 1) (Stamatakis 2014). This phylogenetic relationship is consistent with a previous tree constructed with AFLP markers by Takahashi *et al.* (2016). These phylogenetic relations are important in what follows because in the absence of introgression, *sinensis* and *tymensis* are expected to be more genetically similar to each other than to *pungitius.*

To assess the extent of genetic divergence between the species we estimated the number of synonymous substitutions. For autosomes, the mean number of synonymous substitutions per site (*d*_S_) between *sinensis* and *tymensis* was 0.018, whereas the mean for *sinensis* and *pungitius* was 0.025 (*N *=* *9772 autosomal genes; supplementary fig. S2, Supplementary Material online). For pairwise comparisons between these species and *G. aculeatus*, mean *d*_S_ ranged from 0.126 to 0.129 (supplementary fig. S2, Supplementary Material online).

Gene trees can be used to determine if a chromosome region is an SDR, and if it acts as an XY or ZW system (Toups et al. personal communication). Briefly, consider a young Y chromosome that was established from a unique mutation or introgression event. Within its SDR, all copies in the population are descended from the single ancestral Y. In a gene tree of sex chromosomes sampled from males and females, the Y-linked sequences will form a clade. Consequently, half of the chromosomes sampled from males will form a clade, while the other half of male chromosomes (their Xs) will be intermingled on the tree with those sampled from females. In the PAR, however, recombination reshuffles X- and Y-linked sequences each generation, so monophyly is not expected for the Y-linked sequences. Thus, a gene tree in which half of the chromosomes from males form a monophyletic clade is indicative of an SDR that acts as an XY system. A gene tree with this topology is referred to as “XY-consistent” (Toups *et al.* personal communication).

**Fig. 2.**
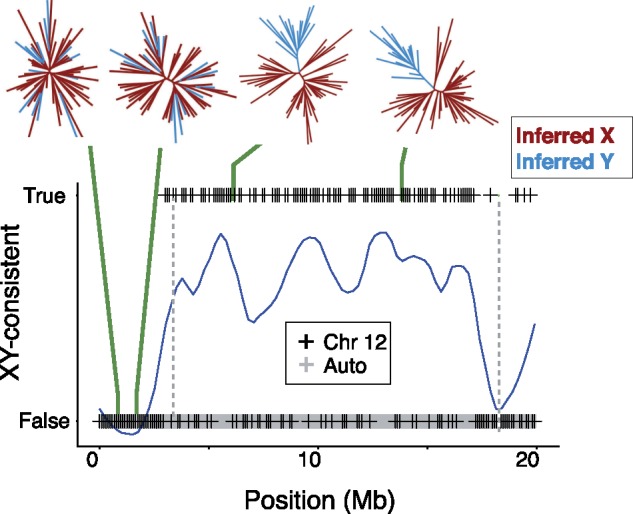
The incidence of XY-consistent topologies on Chr 12 and the autosomes in 100-kb windows in *Pungitius pungitius*. Blue curve traces the loess regression for Chr 12. Vertical dashed lines show the inversion breakpoints. Examples are shown of two gene trees from the PAR that do not have XY-consistent topologies, and two gene trees from the SDR that do. No autosomal gene tree showed an XY-consistent topology. Positions on the *X* axis refer to Chr 12, and autosomes have been rescaled to the same length.

With these expectations in mind, we constructed gene trees in 100-kb windows along Chr 12 (Stamatakis 2014; Cook and Andersen 2017). Unexpectedly, all male sequences, rather than just half of them, cluster together (supplementary fig. S3, Supplemental Material online). This may occur for two reasons. The first is heterozygote dropout, which in some cases will lead to homozygous calls for the Y-linked allele (Li 2011). The second is misassignment by the phasing algorithm. To refine our analysis of gene trees, we assigned heterozygous alleles on Chr 12 in males as either X- or Y-linked (see supplementary file Materials and Methods, Supplementary Material online). Briefly, gene trees were constructed in overlapping sliding windows across the chromosome. For each gene tree, male alleles were scored as Y-linked if they occurred in an exclusively male clade, as expected of Y haplotypes. Because the windows were overlapping, each locus was interrogated with multiple gene trees. The allele that was identified as Y-linked in the greatest number of gene trees was recorded as the Y-linked allele for each sample. After this refinement, inferred Y haplotypes make distinct clusters from the inferred X haplotypes (fig. 2), suggesting that the sex determination system of *pungitius* is an XY system and that there was a single origin of the Y.

### Evidence for Hybridization

If the *pungitius* Y chromosome introgressed from *P. sinensis*, we expect there will be traces of historical gene flow between these two species elsewhere in the genome. We assessed gene flow between all three species in 100-kb windows across the genome using the ${\hat{f}}_{d}$ statistic (Martin *et al.* 2015), which is a modification of the ABBA-BABA statistic (Green *et al.* 2010). In nearly every autosomal window, *sinensis* and *pungitius* share an excess of derived mutations relative to *tymensis* (supplementary fig. S4, Supplementary Material online; mean ${\hat{f}}_{d}$ for all autosomal windows = 0.12; mean ${\hat{f}}_{d}$ for the SDR = 0.22).

The two species have also exchanged mitochondria. As shown previously (Takahashi *et al.* 2016), mitochondrial haplotypes from *sinensis* and *pungitius* are more similar to one another than either is to *tymensis* (supplementary fig. S5, Supplementary Material online). This relationship is discordant with the nuclear phylogeny. This discordance has been attributed to introgression of the mitochondrial genome between *pungitius* and *sinensis* (Takahashi et al. 2016). Hence, nuclear and mitochondrial DNA indicate that *pungitius* and *sinensis* hybridized in the past, which gave opportunity for introgression of the proto-Y chromosome into *pungitius*.

### Introgression of the *Pungitius* Y

**Fig. 3.**
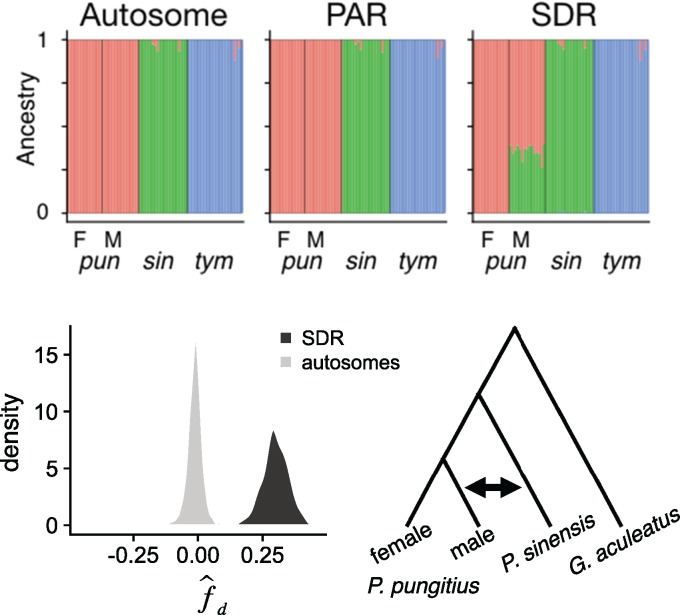
Shared ancestry *P. pungitius* males and *P. sinensis*. (Top): Admixture plots for an autosome (Chr 1), and the PAR and SDR from Chr 12 with female (F) and male (M) *Pungitius pungitius* separated. The three species are genetically distinct in the autosome and PAR. But in the SDR, *P. pungitius* males share about equal ancestry with *P. pungitius* females and *P. sinensis*. The ancestry that males share with females represents the X chromosomes, while that shared with *P. sinensis* represents the Y chromosomes. (Bottom): $\hat{f}$_*d*_ statistic (ABBA-BABA) supports introgression specifically between *P. sinensis* and *P. pungitius* males. $\hat{f}$_*d*_ was calculated for 100-kb windows across the genome, testing for excess of shared derived alleles between *P. sinensis* and *P. pungitius* males relative to *P. pungitius* females. The distribution for windows taken from the SDR (from 4 to 17 Mb) is shown in black (mean = 0.30). The distribution for equivalent regions from the autosomes is shown in gray (mean=−0.01).

Having confirmed previous hybridization between the two species, we turned to the evolutionary history of the *pungitius* sex chromosome. Males, but not females, show high levels of shared ancestry with *sinensis* on Chr 12. Supplementary figure S6, Supplementary Material online shows the results of a principal component analysis (PCA) (Jombart and Ahmed 2011). In the PCA, male *pungitius* cannot be distinguished from females in an autosome (Chr I) or the PAR. In contrast, males show a marked similarity to *P. sinensis* in the SDR. Admixture analysis (Alexander et al. 2009) yielded similar results, with ∼30% ancestry shared between *sinensis* and *pungitius* males within the SDR, but very little in autosomal and PAR regions (fig. 3 and supplementary fig. S7, Supplementary Material online).

To corroborate those results, we again calculated ${\hat{f}}_{d}$, this time testing for excess of derived alleles shared between *sinensis* and *pungitius* males relative to *pungitius* females. Mean ${\hat{f}}_{d}\;$for windows on autosomes was very small (mean = −0.01), but was much larger for windows from the SDR (mean = 0.30) (fig. 3).

**Fig. 4.**
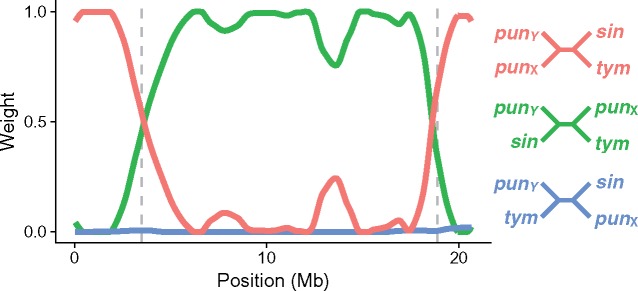
Topology weights for gene trees of 100-kb windows across Chr 12 using inferred X and Y haplotypes. The abbreviations for the species names are given in figure 1. Predicted inversion breakpoints (Natri 2015) are shown by dashed vertical lines. Within the SDR, *Pungitius pungitius* Y and *P. sinensis* haplotypes almost always sisters. In the flanking regions, the *P. pungitius* X and Y are sisters, as expected of recombining chromosomes.

We next examined the phylogenetic history of the *pungitius* Y chromosome. Here, we used gene trees constructed from inferred *pungitius* X and Y haplotypes (see supplemental file Materials and Methods, Supplemental Material online). As before, gene trees were constructed in 100-kb windows across the length of the chromosome (207 gene trees in total; Stamatakis 2014; Cook and Andersen 2017). Within the SDR, *pungitius* Y haplotypes nearly always clustered with *sinensis* haplotypes. We quantify this tendency using the Twisst algorithm (Martin and Van Belleghem 2017). From each full gene tree, subtrees are iteratively sampled, with one branch from each of four designated groups: *pungitius* X, *pungitius* Y, *sinensis*, and *tymensis*. The frequency of each possible topology among these sampled subtrees can be thought of as its proportional weight in the full gene tree. Figure 4 shows that the *pungitius* Y and *sinensis* haplotypes almost always cluster together within the SDR. But in the PAR, *sinensis* clusters with *tymensis*, as expected from their phylogenetic relationship (fig. 1).

There is an alternative to the introgression hypothesis that might explain why *sinensis* is most similar to *pungitius* within the SDR. Incomplete lineage sorting, or ILS, can cause some regions of the genome to have an evolutionary history that is discordant with the species phylogeny (Edwards et al. 2016). Under the ILS hypothesis, the most recent common ancestor of Chr 12 in *pungitius* and *sinensis* would predate the speciation event in which the *pungitius* lineage diverged from the ancestor of *sinensis* and *tymensis*. In that case, the genetic divergence between them is expected to be greater than the genomic average. In contrast, the introgression hypothesis predicts the opposite pattern. Estimates of genetic differentiation based on synonymous substitutions (supplementary fig. S2, Supplementary Material online) and all SNPs together support the introgression over the ILS hypothesis (supplementary figs. S8 and S9, Supplementary Material online; Nei and Li 1979).

**Fig. 5.**
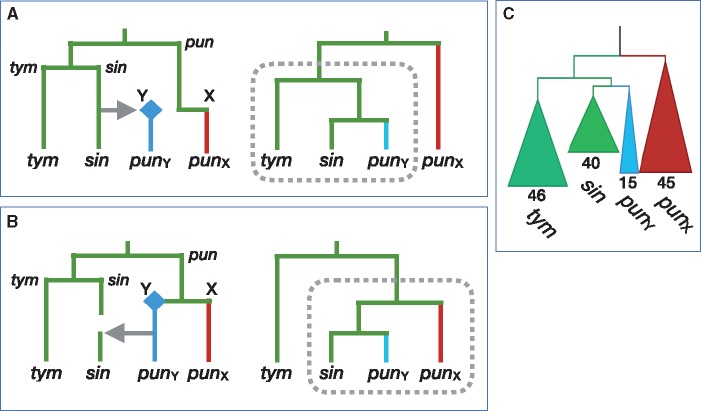
Gene trees predicted under two hypotheses for the direction of introgression. X and Y chromosomes are shown in blue and red, respectively; the origin of a new Y is shown as a diamond. The abbreviations for the species names are given in figure 1. (A) Introgression of Chr 12 from *Pungitius sinensis* into *P. pungitius* creates the *P. pungitius* Y chromosome (left). This generates a gene tree in which those two lineages are sister to *P. tymensis* (right). (B) Introgression of the Y from *P. pungitius* into *P. sinensis* generates a gene tree in which those two lineages are sister to the *P. pungitius* X chromosomes. (C) The gene tree from the SDR of Chr 12 (10–12 Mb) supports hypothesis (A). The numbers indicate the number of sampled chromosomes in each clade.

Next, we investigated the direction of introgression of Chr 12 between *sinensis* and *pungitius*. As shown in figure 5, gene trees again provide the answer. If *pungitius* had donated Chr 12 to *sinensis*, then *sinensis* haplotypes within this region would nest within the *pungitius* chromosomes in the gene tree (fig. 5*B*). But the opposite pattern is observed: the *pungitius* chromosomes nest within *sinensis* (fig. 5*A*). Hence, our data are consistent with the hypothesis that the Y chromosome in *Pungitius pungitius* arose via introgression from *P. sinensis*. It is interesting to note that the direction of introgression for the Y chromosome (from *sinensis* to *pungitius*) contrasts with the direction for the mitochondrial genome inferred by Takahashi et al. (2016) (from *pungitius* to *sinensis*).

Figure 5 also gives us insights into the questions regarding whether Chr 12 in *pungitius* functioned as a Y when it first introgressed, or if it became a sex chromosome later. Had Chr 12 introgressed as an autosome, we expect that it would have become fixed, driving the ancestral *pungitius* Chr 12 homologues to extinction. In this case, all *pungitius* haplotypes would cluster with *sinensis* rather than just the Y. As this is not the case, the chromosome must have functioned as a Y from the moment that it entered the *pungitius* population.

### Evolution of the Y since Its Introgression

**Fig. 6.**
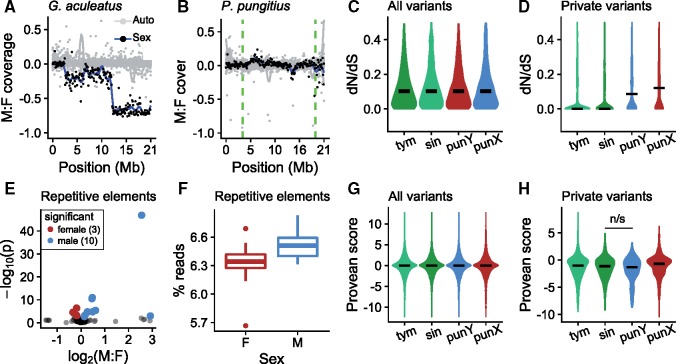
The *Pungitius pungitius* Y shows limited degeneration. (A) Male: female fold coverage for the *Gasterostreus aculeatus* sex chromosome (Chr 19) shows known evolutionary strata. (B) The same plot for the *P. pungitius* sex chromosome (Chr 12) shows no loss of coverage in males. (C) *d*_N_/*d*_S_ for genes on Chr 12 (*N* = 838) do not vary between species when all variants are considered. (D) *d*_N_/*d*_S_ is elevated for both X and Y for variants private to the particular groups. (E) Differences in fold coverage for repetitive elements between males and females. Repetitive elements significantly greater in females are shown in red and those significantly greater in males are shown in blue. (F) Males show a greater overall proportion of reads mapping to repetitive elements (*t* test, *P *<* *0.01). (G) Estimates of the functional effects of amino acid substitutions (Provean scores) show no difference between species when all variants are considered (*N* = 7485–7720 amino acid substitutions on Chr 12). (H) Provean scores for private Y-linked mutations are slightly elevated, but not significantly greater than those in *P. sinensis* (Mann–Whitney *U* test, *P *=* *0.22). Number of private amino acid substitutions were: *P. tymensis* 569, *P. sinensis* 330, *P. pungitius* Y 172, *P. pungitius* X 868.

The *pungitius* Y chromosome shows only subtle signs of degeneration. In contrast to distinct evolutionary strata seen on the *G. aculeatus* sex chromosome (fig. 6*A*; White, Kitano, and Peichel 2015), we detected little difference in the sequencing coverage between male and female *pungitius* anywhere along Chr 12 (fig. 6*B*). This indicates that neither extreme sequence divergence from the *G. aculeatus* reference genome nor extensive deletions have occurred on the *pungitius* Y. Males of *pungitius* do, however, show higher fold coverage for repetitive elements than females (fig. 6*E* and *F*) (Luo et al. 2015; Smit et al. 2015). As the Y chromosome is the only major genomic difference expected between males and females, this pattern is indicative of an accumulation of repetitive elements on the Y.

Analysis of coding sequences also indicated limited degeneration. Because most amino acid changes are removed by purifying selection, the ratio of nonsynonymous substitutions to synonymous substitutions (*d*_N_/*d*_S_) is expected to be less than 1. Elevated *d*_N_/*d*_S_ ratios can be a sign of relaxed purifying selection or positive selection on amino acid substitutions. To test for evidence of relaxed purifying selection on the Y, we calculated *d*_N_/*d*_S_ ratios for *tymensis*, *sinensis*, female *pungitius* (X-linked), and inferred Y-linked substitutions based on pairwise comparisons with *G. aculeatus* (Yang 2007). When considering all substitutions within the SDR, we observed no differences between groups (fig. 6*C*). However, when we considered only substitutions that are private to each group, *d*_N_/*d*_S_ ratios were significantly higher for both X- and Y-linked loci in *pungitius* compared with *sinensis* and *tymensis* (fig. 6*D*). This indicates that both the X and Y in *pungitius* have undergone relatively rapid protein evolution compared with the other two species. For reference, overall pairwise synonymous substitution rates are shown in supplementary figure S2*A*, Supplementary Material online.

We sought evidence of deleterious amino acid substitutions using PROVEAN (Choi et al. 2012; Yoshida et al. 2017). PROVEAN estimates the functional significance of amino acid substitutions based on conservation of the site in homologous sequences from other taxa. Here again, we observed no differences when considering all substitutions (fig. 6*G*). For private substitutions, the *pungitius* Y showed slightly lower Provean scores, predictive of greater deleteriousess, but the difference with *sinensis* was not significant (fig. 6*H*). Similar results were observed when Provean scores were analyzed in a binary fashion, using a recommended cutoff of −2.5 for calling deleterious substitutions (supplementary fig. S10, Supplementary Material online; Choi et al. 2012; Yoshida et al. 2017). While the *d*_N_/*d*_S_ ratios observed for the X-linked loci were relatively high (fig. 6*D*), substitutions on the X tended toward higher Provean scores (predicted to be less deleterious) than those in each of the other three groups (Mann–Whitney *U* test; *P* < 0.05; fig. 6*H*). This indicates that the high rates of amino acid substitution observed on the X may reflect positive selection rather than relaxed purifying selection.

**Fig. 7.**
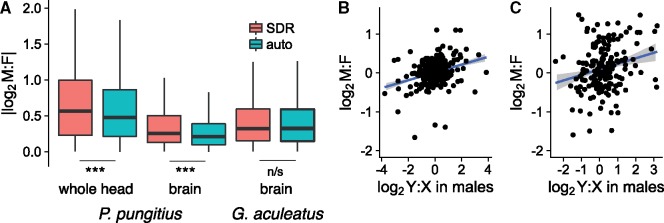
Evolution of transcription on the *Pungitius pungitius* Y chromosome. (A) Differences in expression between sexes are greater for genes in the *P. pungitius* SDR. At right is the ratio for the same genes in the threespine stickleback, where they are autosomal. (Significance tested with Mann–Whitney *U* tests.) To control for potential effect of mapping efficiency, M:F ratios for *P. pungitius* RNA-seq fold coverage were normalized by subtracting the M:F ratio calculated from DNA data. (B) Male:female expression ratios in brain tissue are predicted by Y:X differences within males. (D) Male:female expression ratios whole head tissue correlate with Y:X ratios within males, but less strongly than in brain tissue.

Sex biased gene expression is greater in the *pungitius* SDR than on autosomes (fig. 7*A*; Love et al. 2014; Anders et al. 2015). This is true for expression measured from whole heads and from dissected brains. In *G. aculeatus* on the other hand, where Chr 12 is autosomal, sex biased expression in this region is similar to other autosomes (fig. 7*A*). A possible artifact is that the sex differences in *pungitius* might result from decreased mapping efficiency of Y chromosome reads. However, the sex differences persist after we normalize the male: female expression ratio by subtracting the male:female ratio from DNA reads (fig. 7*A*). Moreover, expression of SDR-linked genes is biased toward males and females roughly equally (supplementary fig. S11*A*, Supplementary Material online). Similar results are seen when we compare expression in male *pungitius* to the ancestral state based on *G. aculeatus* males (supplementary fig. S11*B*, Supplementary Material online). This indicates that the observed sex biased expression cannot be explained merely by loss of transcription from the Y due to degeneration, nor reduced mapping efficiency of Y-linked reads.

Differences between male and female expression appeared to be driven at least in part by changes in transcription of the Y relative to the X. Analysis of allele-specific expression (Krueger and Andrews 2016) found that Y:X expression ratios in male brain tissue are significantly correlated with male:female ratios (*R*^2^ = 0.09; fig. 7*B*). A similar but less pronounced relationship is observed for expression in whole heads (*R*^2^ = 0.05; fig. 7*C*). This shows that evolution of transcription on the Y has contributed to overall differences in male female gene expression.

## Discussion

Our whole genome resequencing corroborates the hypothesis that a new Y chromosome in the ninespine stickleback, *P. pungitius*, was established by introgression from the Amur stickleback, *P. sinensis* (Natri 2015). Genetic differentiation between males and females (fig. 1) and gene trees (fig. 2) in a region spanning roughly 15 Mb on Chr 12 show there is blocked recombination between the X and Y chromosomes. This region is the putative sex determining region (SDR). Admixture and introgression analyses indicated gene flow occurred in the past between *pungitius* and *sinensis*, and the greatest introgression in the genome occurred in the SDR (supplementary figs. S4, S5, and S9, Supplementary Material online). Analyses of gene trees confirm the *pungitius* Y is more closely related to Chr 12 of *sinensis* than to the X chromosome of *pungitius*. The direction of introgression was from *sinensis* into *pungitius*. The data strongly suggest that Chr 12 acted as a Y from the moment that it introgressed, rather than evolving into a sex chromosome later, because the *pungitius* X is derived from the ancestral *pungitius* autosome rather than the chromosome that introgressed from *sinensis*.

This leaves open the question of when the masculinizing mutation on the *pungitius* Y was acquired. Yet another intriguing observation made by Natri (2015) is that a dominant feminizing locus from Chr 7 is fused to Chr 12 in *sinensis* (Natri 2015). This suggests a hypothesis for the dominance relations between the sex determining factors. In *sinensis*, which has a ZW system, the feminizing W chromosome is dominant to the Z. The simplest hypothesis is that it was a *sinensis* Z that introgressed into *pungitius*, where it acted as a Y that was dominant over the resident homologues, which then served as X chromosomes. Disappointingly, we did not detect substantial genetic divergence between *sinensis* males and females on Chr 7 or 12, so we are not able to verify that scenario. This lack of divergence in *sinensis* suggests that its sex determining region is small and/or has not experienced suppressed recombination for very long.

Figure 5*A* shows the historical scenario that is the simplest explanation for all of our results, but alternative hypotheses can be considered. For instance, assume that the ancestor of all three species had an XY system on Chr 12. The data then could be explained if there was a transition to ZW sex determination in the ancestor of *sinensis* and *tymensis* after it diverged from *pungitius*, followed by an introgression between *pungitius* and *sinensis* (supplementary fig. S12*A*, Supplementary Material online). The evidence of a ZW locus fused to Chr 12 in *sinensis* (Natri 2015) is consistent with a transition from a XY to a ZW system. However, genetic divergence between the X in *pungitius* and Chr 12 in other two species does not support this hypothesis. If the XY system on Chr 12 is ancestral, then the *pungitius* X is expected to be more genetically diverged from *sinensis* and *tymensis* than the genomic average (because divergence of the sex chromosomes preceded the speciation event). Instead, the distributions of genetic divergence for the SDR and the autosomes are largely overlapping (supplementary fig. S13, Supplementary Material online). For these reasons, we favor the hypothesis that the XY sex determination system on Chr 12 in *pungitius* was established by introgression from the *sinensis* lineage (fig. 5*A*). We are not, however, able to say what type of sex determination system *pungitius* had before that event.

Many species have a large SDR over which recombination between the X and Y is suppressed (Bachtrog et al. 2014). Typically, recombination suppression is thought to occur when one or more inversions fix on either the X or Y only after the sex chromosomes were established (Charlesworth et al. 2005). Although they are often thought to occur on the Y, inversions can be favored on either the proto-Y or the proto-X (Charlesworth et al. 2005). The genomic history of *Pungitius* sticklebacks shows there is a second and very different pathway to the evolution of a large nonrecombining SDR. As reported by Natri (2015), the X chromosome in *pungitius* carries a large inversion relative to the ancestral state found in *sinensis*, *tymensis*, and the *pungitius* Y chromosome. Hence, within *sinensis*, that region of Chr 12 recombined at a normal rate. But from the moment that the new chromosome introgressed from *sinensis*, the inverted region of the *pungitius* Chr 12 (now acting as a X) was blocked from recombining with its introgressing homolog (now acting as an Y). We expect the *pungitius* Y will follow the road of degeneration that seems inevitable for nonrecombining sex chromosomes (Bachtrog 2006). But at the time it first became established, the large SDR of the *pungitius* Y was likely genetically fully functional, and much more typical of an autosome than a Y chromosome in its structure.

The young Y chromosome in *pungitius* joins several other examples of sex chromosomes that have introgressed from other species (Tosi et al. 2002; Stölting et al. 2013; Corcoran et al. 2016). These cases defy the general rule that sex chromosomes pass less easily between species than do autosomes (Muirhead and Presgraves 2016; Payseur and Rieseberg 2016). X and Z chromosomes introgress less in hybrid zones than do autosomes in many taxa, for example, in birds (Saetre et al. 2003; Bronson et al. 2005; Carling and Brumfield 2008; Storchová et al. 2010), butterflies (Hagen and Scriber 1989), fishes (Ravine et al. 2018), and mammals (including humans) (JanouŠek et al. 2012; Carneiro et al. 2014; Sankararaman et al. 2014). This bias is generally attributed to the preferential accumulation of hybrid incompatibilities on X and Z chromosomes, the so-called “large X effect” (Charlesworth et al. 1987; Coyne and Orr 1989, 2004; Presgraves 2008). Sex-biased dispersal may also play a role in some taxa (Petit and Excoffier 2009). Based on the very limited number of examples, it seems that introgression of Y and W chromosomes is even more restricted than X and Z chromosomes. The most obvious explanation comes from Haldane’s rule, the observation that in hybrids the heterogametic sex suffers sterility or inviability more often than the homogametic sex (Coyne and Orr 1989, 2004). As we noted in the introduction, male (but not female) hybrids between *pungitius* and *sinensis* are sterile (Takahashi et al. 2005). This situation precludes the introgression of a Y chromosome between contemporary populations. We suggest that introgression of the *pungitius* Y occurred at a time when (or in populations where) genetic isolation between the two species was weaker.

In ZW systems, several cases have been reported in which it appears mitochondria introgressed by positive selection (Toews and Brelsford 2012; Irwin 2018). These cases presumably also involve introgression of the W chromosomes, since they are maternally coinherited with the mitochondria. In these cases, further evidence is needed to determine whether it was selection on the mitochondrion or the W that caused them both to introgress.

What then might have driven the *sinensis* chromosome 12 to introgress into *pungitius*? Drift seems implausible because the SDR of the Y introgressed, but the PAR regions of the same chromosome did not. Moreover, the *pungitius* autosomes also show very little evidence of admixture from *sinensis* (supplementary fig. S7, Supplementary Material online). That pattern suggests roles for selection: positive selection that favored the invasion of the SDR, and negative selection on autosomal loci, as well as the two PAR regions that prevented them from hitchhiking along. Any type of natural or sexual selection advantage of the novel SDR could have been involved. One intriguing possibility, similar to the hypothesis described by Corcoran et al. (2016), is that the introgressed chromosome was favored because of extensive degeneration of the previous sex chromosome.

Alternatively, the new Y might have spread into *pungitius* by meiotic drive (Úbeda et al. 2015). It is becoming increasingly evident that drive systems are common in many, if not most, species (Lindholm et al. 2016), and may critically influence the evolution sex chromosomes (Abbott et al. 2017; O’Neill and O’Neill 2018). Once introduced into the *pungitius* genetic background, a driving allele might have escaped from unlinked repressors that had evolved in *sinensis* to suppress its driving action. Preferential introgression of a sex chromosomes over autosomes can also result from a dominance drive linked to Darwin’s corollary (Sciuchetti et al. 2018).

It should be noted that several examples of autosomal “supergenes” seem to have similar histories of introgression (Schwander et al. 2014). These include a very large chromosomal region controlling social organization in the fire ant (Wang et al. 2013), another that mediates sexual behavior in white-throated sparrows (Tuttle et al. 2016; Sun et al. 2018), and yet another that is responsible for wing color patterning in *Heliconius* butterflies (Jay et al. 2018). Our results expand this list, and show that semiporous species boundaries can trigger the evolution of a new sex chromosome.

The Y chromosome appears to have entered *pungitius* very recently, as it shows only subtle signs of degeneration. Several mechanisms cause nonrecombining regions of Ys to accumulate deleterious mutations (Bachtrog 2013), and can eventually lead to deletion of large chromosomal regions (Charlesworth et al. 2005). Males in our sample do not show greatly decreased mapping efficiency, indicating that large-scale deletions have not yet occurred on the Y. It does, however, show accumulation of repetitive elements and elevated *d*_N_/*d*_S_ ratios. Marginal elevation of predicted deleteriousness among new Y-linked amino acid substitutions suggests that the higher *d*_N_/*d*_S_ ratios reflect relaxed purifying selection. These subtle signs of degeneration are similar to those described in the young neo-Y chromosome in the Japan Sea stickleback (Yoshida et al. 2014). Here also, no large-scale gene deletions or frame shifts are apparent, but the neo-Y has accumulated higher density of deleterious mutations than the X (Yoshida et al. 2017). The accumulation of repetitive elements may contribute to previous observations that the Y in pungitius is heteromorphic, and considerably larger than the X (Ross et al. 2009).

Despite its recent origin, the sex chromosome in *pungitius* had evolved elevated levels of sex-biased gene expression. This did not appear to be due to degeneration of expression from the Y, as males did not show greater loss of expression from SDR-linked genes relative to females or to their ancestral state (inferred from *G. aculeatus* males). Evidence suggests that sex-biased expression of genes on the sex chromosomes is driven by regulatory evolution on the Y. Hence some of the earliest evolutionary changes apparent in this young sex chromosome are the accumulation of repetitive elements, higher prevalence of amino acid substitutions, and changes in transcription.

## Materials and Methods

For detailed explanation of materials and methods, see supplementary file Materials and Methods, Supplementary Material online.

### Data Accessibility

Sequencing data generated for this project are archived on the NCBI SRA database (Accession SRP151119). Software used for this study included: VCFtools (Danecek et al. 2011) Genome Analysis Toolkit (DePristo et al. 2011); adegenet (Jombart 2008); Bowtie2 (Langmead and Salzberg 2012); BWA (Li and Durbin 2009); cutadapt (Martin 2011); Bedtools (Quinlan and Hall 2010); Scripts used for data processing and analysis, along with intermediate data files are available on GitHub: https://github.com/grovesdixon/pungitius_sex_chromosome; last accessed October 3, 2018 (https://zenodo.org/badge/latestdoi/140638093; last accessed October 3, 2018).

## Supplementary Material

Supplementary data are available at *Molecular Biology and Evolution* online.

## Supplementary Material

Supplementary Data
