# Supplementary material for: The Origin of a New Sex Chromosome by Introgression between Two Stickleback Fishes
Source: Mol Biol Evol. 2018 Oct 1;36(1):28–38. doi: 10.1093/molbev/msy181 (PMC6340465; doi:10.1093/molbev/msy181)
Supplement: Supplementary Data [file msy181_supp.zip › Materials and Methods.pdf]

Materials and Methods for

The origin of a new sex chromosome by introgression between two stickleback fishes

Groves Dixon[1]

Jun Kitano[2]

Mark Kirkpatrick[1]

[1]Department of Integrative Biology, University of Texas, Austin, Texas, United States of America

[2]Division of Ecological Genetics, Department of Population Genetics, National Institute of Genetics, Mishima, Shizuoka, Japan

Corresponding Author:

Groves Dixon

grovesdixon@gmail.com

Materials and Methods

Sampling

Pungitius pungitius was collected from the Biwase tidal pond in Hamanaka, Japan, in April 2017 (15 females and 15 males). *P. tymensis* was collected from Monshizu Pond in Akkeshi, Japan, in November 2016 (11 females and 15 males). *P. sinensis* was collected from the Hatsume River in Yuza, Japan in August 2016 and March 2017 (9 females and 13 males). Sex was determined by inspecting the gonads under a dissecting microscope.

Library preparation and sequencing

Genomic DNA was isolated with DNeasy Blood & Tissue Kit (Qiagen, Hilden, Germany). Genomic libraries were constructed using NEBNext Ultra DNA Library Prep Kit for Illumina (NEB, Ipswich, MA, USA) and NEBNext Multiplex Oligos for Illumina (Dual Index Primers). Each fish received a unique barcode. After quantifying DNA concentrations with the Library Quantification Kit (Takara, Shiga, Japan), libraries were pooled such that the DNA from each fish had the same molar concentration. Sequencing was performed on an Illumina HiSeq 4000 at the Genome Sequencing and Analysis Facility at the University of Texas, Austin. Sequencing of the *Pungitius* libraries produced an average of 12.9 million 150 bp paired-end reads per individual (mean = 8.3x raw coverage across all samples). Raw coverages for the individual species were as follows: *P. pungitius* 7.3x; *P. sinensis* = 9.7x; *P. tymensis* = 8.4x. To provide an outgroup for phylogenetic analyses, we downloaded sequences for *G. aculeatus* and *G. nipponicus* from the DNA Databank of Japan (Accession number DRA001136) which were published previously (Yoshida et al. 2014).

Genotyping

Adapter sequences were trimmed using cutadapt (Martin 2011). Reads were mapped to the *G. aculeatus* reference genome (Glazer et al. 2015) using the BWA –mem algorithm (Li and Durbin 2009). PCR duplicates were removed using Picard (https://broadinstitute.github.io/picard/). Mean duplication rate was 22%. SNPs were called using mpileup (Li 2011). Genotypes with quality scores below 20 were excluding using bcftools. Indels, singletons, and sites with more than two alleles were removed using VCFtools (Danecek et al. 2011). Mean coverage for the final filter-passing set of SNPs was 2.24 reads. Overall patterns of genetic variation were assessed using principal component analysis implemented in the R package agenet (Jombart 2008). Based on these analyses, two individuals from *sinensis* were removed because they did not cluster with the rest of the individuals in the species. For comparisons between males and females within species (fig. 1), variants were filtered with a minimum minor allele frequency of 0.1 and a maximum of 10% missing genotypes. For phylogenetic analyses, SNPs were filtered to maximum of 10% missing genotypes among samples from all species.

Comparison of males and females

Comparisons between males and females were made in 100 Kb sliding windows. F_{ST} was calculated as in Weir and Cockerham (1984) using VCFtools (Danecek et al. 2011). Nucleotide diversity, (π) was also calculated using VCFtools. Fold coverage for males and females was calculated using Bedtools (Quinlan and Hall 2010), and compared using DESeq2 (Love et al. 2014).

Assessment of historical gene flow

Gene flow between *pungitius* and *sinensis* was assessed in several ways. Principal component analysis was performed using Adgenet (Jombart and Ahmed 2011). Estimation of the extent of shared ancestry was performed using Admixture (Alexander et al. 2009) with *K* set to three. The degree of introgression was estimated for 100 Kb windows using the $\hat{f}_d$ statistic (Martin et al. 2015).

Gene trees and phylogenetic analyses

Gene trees were estimated using computationally phased SNPs. Phasing was performed for each species separately using Beagle with 10 iterations (Browning and Browning 2007). SNPs were divided into 100 Kb windows using VCFtools and output into fasta format using vcf-kit (Cook and Andersen 2017). Gene trees were constructed with RAxML using the GTRCAT model of mutation (Stamatakis 2014). The species phylogeny was estimated with all autosomal SNPs using the GTRCAT model in RAxML with 1000 bootstrap iterations.

Assignment of Y-linked alleles

As described in the main text, Y-linked alleles can be identified phylogenetically, because they are expected to form a monophyletic clade separate from X chromosomes. We took advantage of this expectation to identify Y haplotypes using computational phasing and gene trees. First, we computationally phased male and female genotypes using Beagle. This provided two haplotypes for each individual, which we labeled arbitrarily as A and B.

The next step was to identify which of the two haplotype in each male represented his Y chromosome. This was done by building gene trees with all male and female haplotypes from windows of Chr 12. For a given gene tree, the Y chromosome clade was identified as the smallest possible monophyletic group composed exclusively of males, and containing at least one haplotype from each male. If no such clade was found, the criteria were relaxed, and the largest exclusively male clade with one haplotype from at least 75% of male individuals was identified as the Y chromosome. Otherwise no Y clade was identified for the gene tree. In this way, haplotypes showing evidence of Y-consistent topologies were identified.

This process was performed for series of overlapping gene trees of varying window sizes. Window sizes were 2000, 1000, 500, and 100 SNPs, with a slide distance of 500, 250, 100, and 25 SNPs respectively. These sizes and slide distances produced 1786, 3576, 8944, and 35790 gene trees respectively. Windows were delineated in SNPs to provide greater precision at regions of high SNP density. Because the windows overlapped, each region of the chromosome was interrogated with multiple gene trees. For each heterozygous male genotype, the Y-linked allele determined by the allele that was assigned to a Y-chromosome clade in the greatest number of gene trees. In the case of a tie, an allele was randomly selected. This included loci for which no Y-consistent clades were observed. Because smaller window sizes result in a greater number of gene trees across the chromosome, they were effectively weighted more heavily than that larger windows. This gives greater weight to the spatially localized scale at which Beagle is likely to be most accurate (Browning and Browning 2007). In regions where the small windows failed to resolve XY haplotypes, Y-assignment was still possible based on signal from larger windows.

Genetic divergence

We estimated genetic divergence between populations of phased haplotypes to infer relative divergence times. These included the relative difference in allele frequency, measured by F_{ST} (Holsinger and Weir 2009), and the average number of pairwise differences per site between populations, measured by d_{xy} (Nei and Li 1979). Note that that the 'xy' subscript in d_{xy} refers to the two populations being compared, not to sex chromosomes. In addition, we developed a third statistic, $\tilde{d}$ (see below).$\tilde{d} = d_{xy}$-(π_Y-π_s)

Comparison of divergence between sex chromosomes and autosomes is complicated by both biological and bioinformatic issues. Y-linked sites are passed on exclusively through male meioses, so they are expected to have higher mutation rates than autosomal sites (Li et al. 2002). Heterozygote dropout and phasing could further bias estimates of divergence. Under the introgression hypotheses, phasing errors will introduce X-linked alleles into Y sequences, increasing the apparent divergence of the *pungitius* Y from Chr 12 of *sinensis* relative to what is seen in autosomes. Further, increased male mutation will increase the nucleotide diversity of the inferred Y-linked haplotypes.

We devised the following statistic to correct for these biases:

$$\tilde{d} = d_{xy} - (\pi_Y - \pi_s) \,,$$

where π_Y is the molecular diversity on the *pungitius* Y and π_s is the diversity at the homologous sites in *sinensis*. The difference $(\pi_Y - \pi_s)$ represents the inflation in d_{xy} that results from higher diversity on the *pungitius* Y caused both by increased mutation rate on the Y and by phasing

errors. In this way, we accounted for both biological factors and bioinformatic artifacts that would inflate the apparent divergence of sex-linked haplotypes.

Repetitive element enrichment

To test for enrichment of repetitive elements on the Y chromosome, we compared fold coverage between male and female reads mapped to repetitive elements extracted from the *G. aculeatus* reference genome and a *de novo* assembly made from the *pungitius* reads. The *de novo* assembly was performed using Soap Denovo version 2.04 (Luo et al. 2015). Assembly was performed using all read files from both sexes with a target genome size of 450 Mb, maximum K-mer size 63, merge level of 3, allowed gap length difference of 250, insert upper bound (b*average insert) set to 3, and the option to keep weakly connected contigs set to "YES". We then used RepeatModeler (Smit et al. 2015) to identify repetitive DNA in both the *G. aculeatus* reference and the *de novo* assembly. Reads from male and female *pungitius* were mapped with Bowtie2 to the concatenated repetitive sequences identified from the two references (Langmead and Salzberg 2012) in single end mode using the --local argument. Fold coverage for reads mapping to each repetitive sequence was counted using bedtools (Quinlan and Hall 2010). Significance testing for differences in fold coverage between male and female *pungitius* was performed with DESeq2 (Love et al. 2014). The size factors used for normalization of the counts in DESeq2 were calculated from read counts against the entire genome. Significance of the difference between males and females in proportion of reads that mapped to repetitive elements was tested using Welch two sample t-test.

Nonsynonymous and synonymous substitution rates

We estimated nonsynonymous (d_N) and synonymous (d_S) substitution rates. First, we identified the most frequent allele for each site in *sinensis*, *tymensis*, *pungitius* females, and the inferred Y chromosomes from *pungitius* males. We then exported fasta files with the variants inserted into the *G. aculatus* reference genome using FastaAlternateReferenceMaker from the Genome Analysis Toolkit (DePristo et al. 2011) and isolated annotated coding sequences. Substitutions per site were estimated based on pairwise comparisons with the *G. aculeatus* reference using PAML (Yang 2007). This analysis was initially performed using all variants found in each group. Most of these, however were shared across all four groups. To better assess substitution rates on the *pungitius* Y after it introgressed from *sinensis*, we repeated the analysis using only private variants, i.e. those found exclusively in one of the four groups. These were assumed to represent mutations that occurred in each group after its most recent common ancestor among the other groups. Significance for differences in d_N/d_S ratios across genes was tested using Mann-Whitney U tests.

Detecting deleterious amino acid substitutions

We estimated the deleterious effects of amino acid substitutions using PROVEAN (Choi et al. 2012). PROVEAN estimates the functional significance of amino acid substitutions based on conservation of the site in homologous sequences from other taxa. Amino acid changes at highly conserved sites are predicted to have greater deleterious effect. Homologous sequences are gathered using Blast against the NCBI NR protein database. Each substitution is given a score estimating its functional significance, with a suggested cutoff of -2.5 to identify

deleterious mutations (Choi et al. 2012; Yoshida et al. 2017). As with our substitution rate estimates, we calculated PROVEAN scores for all amino acid substitution relative to the *G. aculeatus* reference in *sinensis*, *tymensis*, *pungitius* females, and the inferred Y chromosomes from *pungitius* males, and amino acid changes private to each group. Significance of differences in PROVEAN scores was tested with Mann-Whitney U tests.

Gene expression analysis

We evaluated evolution of gene expression on the new Y chromosome using published RNA-seq datasets downloaded from NCBI Short Read Archive (SRA). Data were acquired from two studies. The first concerned the evolution of gene expression on the *G. aculeatus* sex chromosome (White et al. 2015; NCBI SRA accession: PRJDB3147). From this study, we acquired RNA-seq reads generated from whole brain tissue of *G. aculeatus* and *pungitius*, with N=3 replicates per sex from each species. The *G. aculeatus* samples were progeny of wild-caught fish collected in the Bekanbeushi River, Akkeshi, Japan. The *pungitius* were lab-reared offspring of a single male and female collected from the tide pool in Biwase, Japan (White et al. 2015). The second study concerned gene expression responses to organic pollutants (von Hippel et al. 2018; NCBI SRA accession: SRP065970). From this study, we acquired RNA-seq reads generated from full heads of *pungitius*, cut directly posterior to the pectoral fins. Sample sizes were N=8 females and N=5 males. The individuals were wild caught from three locations on St. Lawrence Island, Alaska, in total 8 females and 5 males (von Hippel et al. 2018).

Reads from both studies were trimmed of adapter sequences using cutadapt. Mapping was performed with Bowtie2 (Langmead and Salzberg 2012) using the --local algorithm. The

number of reads mapping to each gene was counted using HTSeq (Anders et al. 2015). Statistical analyses of fold coverages were performed using DESeq2 (Love et al. 2014). Genes with mean read count less than 2 were excluded from the analysis. Significance of differences in the male:female expression ratios between groups of genes was assessed using Mann-Whitney U tests.

To assess the extent to which the evolution of gene expression on the Y chromosome influenced male:female expression differences, we estimated allele-specific expression. First, we called SNPs from the RNA-seq data using mpileup as indicated above. We then identified candidate Y-linked alleles that were absent from females, and heterozygous in at least 80% of males. This 80% cutoff was chosen because it maximized the number of candidate Y-linked alleles relative to false positives. False positives were identified as Y-linked alleles called on autosomes. These SNPs were used to confirm that the Alaskan samples showed male:female genetic divergence similar to that observed for the Japanese populations (supplementary fig. S1, Supplementary Material online). The candidate Y linked alleles were then N-masked in the reference genome using SNPsplit_v0.3.2 (Krueger and Andrews 2016). RNA-seq reads were remapped to the N-masked reference and SNPsplit was used to sort alignments identified to contain either X or Y-linked alleles. When remapping, Bowtie2 was run in end-to-end mode, as required by SNPsplit. Fold coverages for the allele-specific alignments were counted as before using HTSeq. Differences between X and Y-linked expression in males were analyzed using DESEq2. Genes with mean read count less than 1 were not considered.

References

Alexander DH, Novembre J, Lange K. 2009. Fast model-based estimation of ancestry in unrelated individuals. *Genome Res.* 19:1655–1664.

Anders S, Pyl PT, Huber W. 2015. HTSeq-A Python framework to work with high-throughput sequencing data. *Bioinformatics* 31:166–169.

Browning SR, Browning BL. 2007. Rapid and accurate haplotype phasing and missing-data inference for whole-genome association studies by use of localized haplotype clustering. *Am. J. Hum. Genet.* 81:1084–1097.

Choi Y, Sims GE, Murphy S, Miller JR, Chan AP. 2012. Predicting the functional effect of amino acid substitutions and indels. *PLoS One* 7.

Cook DE, Andersen EC. 2017. VCF-kit: Assorted utilities for the variant call format. *Bioinformatics* 33:1581–1582.

DePristo M a, Banks E, Poplin R, Garimella K V, Maguire JR, Hartl C, Philippakis A a, del Angel G, Rivas M a, Hanna M, et al. 2011. A framework for variation discovery and genotyping using next-generation DNA sequencing data. *Nat. Genet.* 43:491–498.

Jombart T, Ahmed I. 2011. adegenet 1.3-1: New tools for the analysis of genome-wide SNP data. *Bioinformatics* 27:3070–3071.

Krueger F, Andrews SR. 2016. SNPsplit: Allele-specific splitting of alignments between genomes with known SNP genotypes. *F1000Research* 5:1–14.

Langmead B, Salzberg SL. 2012. Fast gapped-read alignment with Bowtie 2. *Nat. Methods* 9:357–359.

Love MI, Huber W, Anders S. 2014. Moderated estimation of fold change and dispersion for

RNA-Seq data with DESeq2. *Genome Biol.* 15:1–21.

Luo R, Liu B, Xie Y, Li Z, Huang W, Yuan J, He G, Chen Y, Pan Q, Liu Y, et al. 2015. SOAPdenovo2: an empirically improved memory-efficient short-read de novo assembler. *Gigascience* 4:30.

Martin SH, Davey JW, Jiggins CD. 2015. Evaluating the use of ABBA-BABA statistics to locate introgressed loci. *Mol. Biol. Evol.* 32:244–257.

Nei M, Li W-H. 1979. Mathematical model for studying genetic variation in terms of restriction endonucleases. *Proc. Natl. Acad. Sci. U. S. A.* 76:5269–5273.

Quinlan AR, Hall IM. 2010. BEDTools: A flexible suite of utilities for comparing genomic features. *Bioinformatics* 26:841–842.

Smit A, Hubley R, Green P. 2015. RepeatMasker Open-4.0.

Stamatakis A. 2014. RAxML version 8: A tool for phylogenetic analysis and post-analysis of large phylogenies. *Bioinformatics* 30:1312–1313.

von Hippel FA, Miller PK, Carpenter DO, Dillon D, Smayda L, Katsiadaki I, Titus TA, Batzel P, Postlethwait JH, Buck CL. 2018. Endocrine disruption and differential gene expression in sentinel fish on St. Lawrence Island, Alaska: Health implications for indigenous residents. *Environ. Pollut.* 234:279–287.

White MA, Kitano J, Peichel CL. 2015. Purifying selection maintains dosage-sensitive genes during degeneration of the threespine stickleback y chromosome. *Mol. Biol. Evol.* 32:1981–1995.

Yang Z. 2007. PAML 4: Phylogenetic analysis by maximum likelihood. *Mol. Biol. Evol.* 24:1586–1591.

Yoshida K, Makino T, Yamaguchi K, Shigenobu S, Hasebe M, Kawata M, Kume M, Mori S, Peichel CL, Toyoda A, et al. 2014. Sex chromosome turnover contributes to genomic divergence between incipient Stickleback species. *PLoS Genet.* 10.

Yoshida K, Makino T, Kitano J. 2017. Accumulation of Deleterious Mutations on the Neo-Y Chromosome of Japan Sea Stickleback (*Gasterosteus nipponicus*). *J. Hered.* 108:63–68.
