# Supplementary material for: The Origin of a New Sex Chromosome by Introgression between Two Stickleback Fishes
Source: Mol Biol Evol. 2018 Oct 1;36(1):28–38. doi: 10.1093/molbev/msy181 (PMC6340465; doi:10.1093/molbev/msy181)
Supplement: Supplementary Data [file msy181_supp.zip › supplemental_figures.pdf]

Supplementary Figures

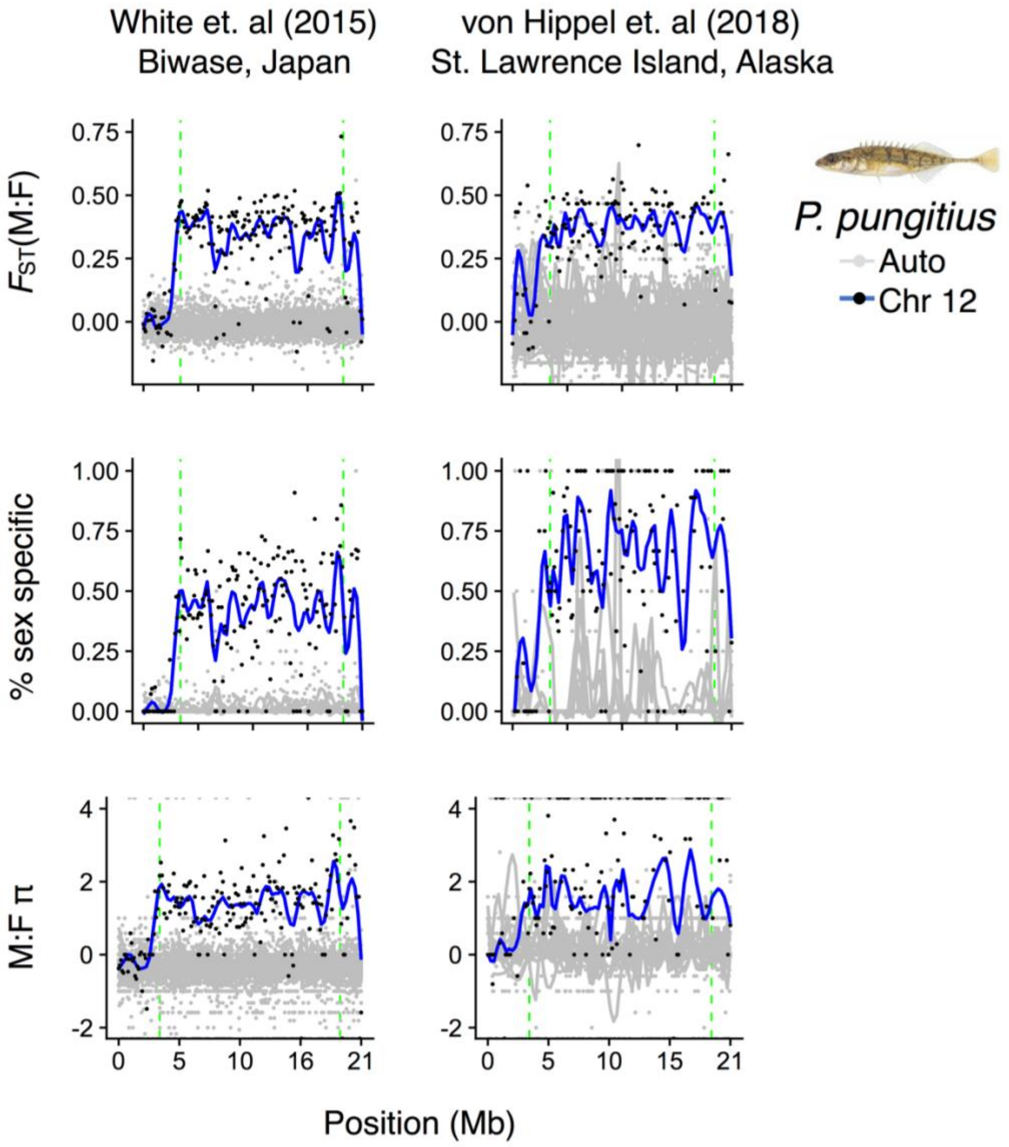

Figure S1: Confirmation of SDR using publicly available RNA-seq datasets. Points represent 100 Kb windows. Data from Chr 12 are shown as black points with a blue loess regression; data from all other chromosomes are in grey. The vertical dashed lines show the inversion breakpoints at 3.5 and 18.9 Mb as identified by Natri et al. (2015). Chromosome positions refer to the sex chromosome, and autosomes have been rescaled to the same length. Both datasets demonstrate evidence of sex chromosomes similar to that observed for our DNA dataset.

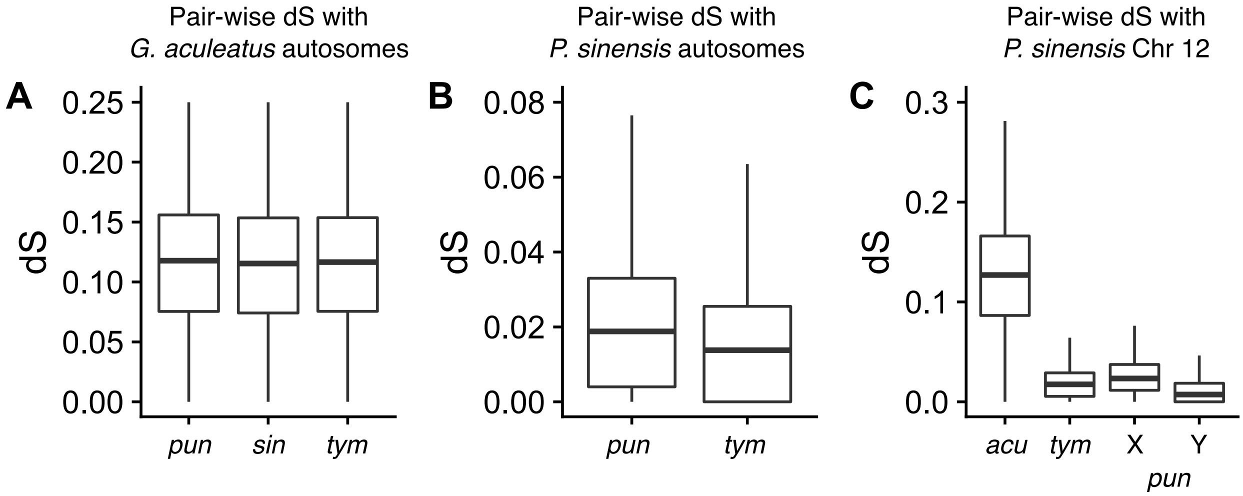

Figure S2: Pairwise estimates of the number of synonymous substitutions per site (dS) between focal species. (A) Pairwise dS estimates compared to *G. aculeatus* (N=9772 genes excluding those on Chr 12). (B) Pairwise dS compared to *P. sinensis* (N=9772 genes excluding those on Chr 12). (C) Pairwise dS compared to *P. sinensis* for genes on Chr 12 (N=838). As expected under introgression hypothesis, pairwise dS for the *P. pungitius* Y (mean = 0.013) was significantly lower than *tymensis* (mean = 0.022; Mann-Whitney U test $p < 0.001$).

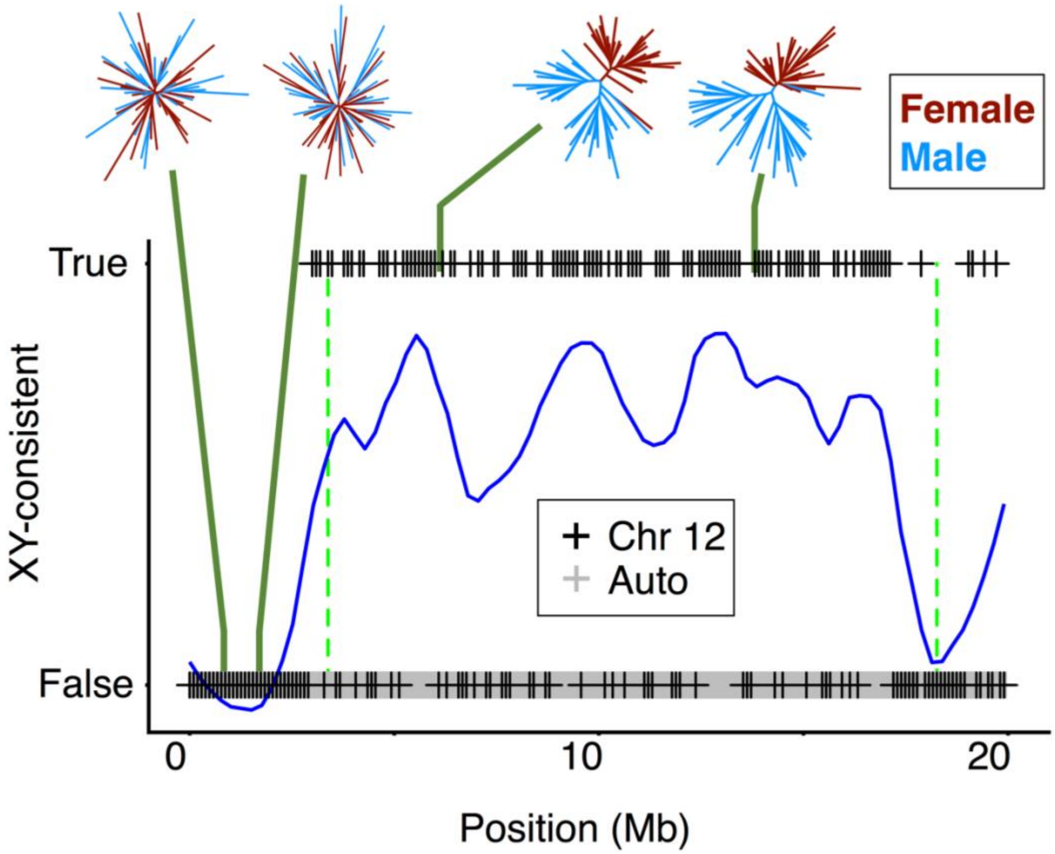

Figure S3: The incidence of XY-consistent topologies on Chr 12 and the autosomes in 100 kb windows. This figure is equivalent to fig. 2 in the main text, with the exception of the example topologies shown at the top. These show the topologies observed prior to Y-chromosome inference, and are color coded by male and female. Note that contrary to expectation, all male haplotypes form single clades within the SDR.

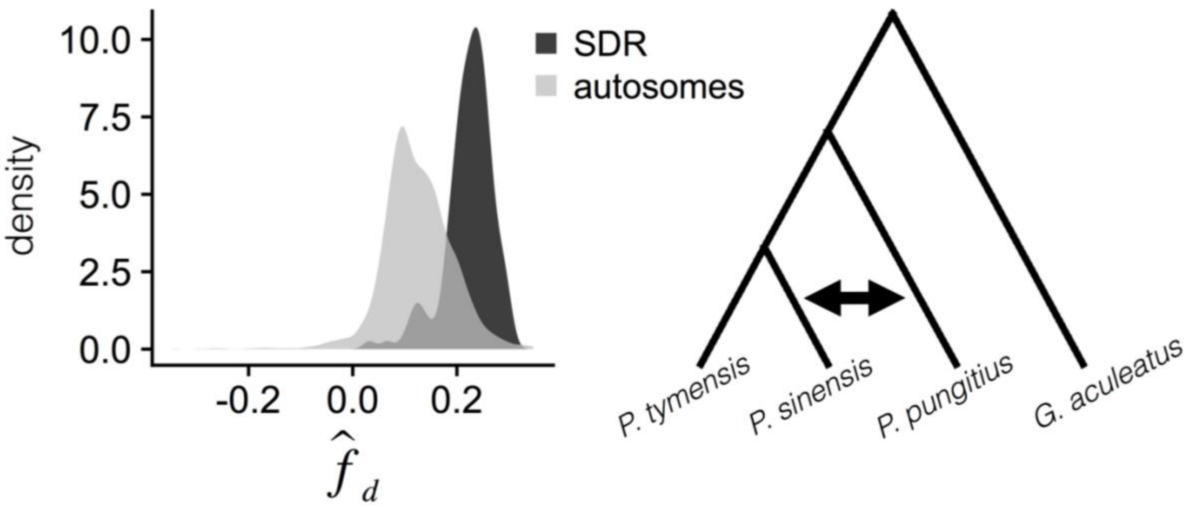

Figure S4: $\hat{f}_d$ statistic (ABBA-BABA) supports historical gene flow between *P. pungitius* and *sinensis*. $\hat{f}_d$ was calculated for 100 Kb windows across the genome, testing for excess of shared derived alleles between *P. pungitius* and *P. sinensis* relative to *P. tymensis*. The distribution for windows taken from the SDR (from 4 - 17Mb) is shown in black (mean = 0.22). The distribution for equivalent regions from the autosomes is shown in grey (mean = 0.12). Both sets of windows were almost all greater than 0, consistent with historical gene flow between *P. pungitius* and *P. sinensis*.

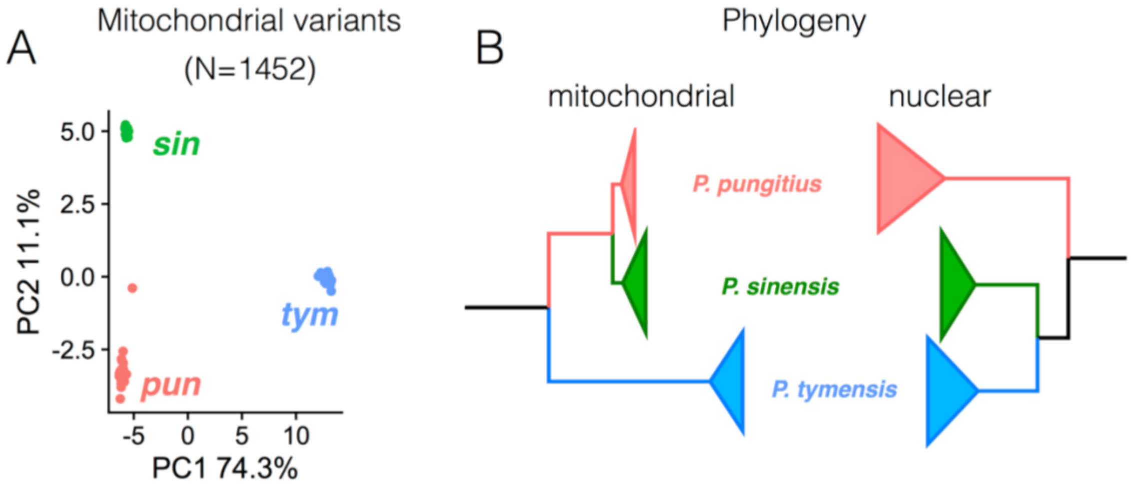

Figure S5: Similarity between *P. pungitius* and *P. sinensis* suggests mitochondrial capture. (A) Principal component analysis based on all mitochondrial SNPs. The first component separates *P. tymensis* from *P. sinensis* and *P. pungitius*. (B) Discordance between the mitochondrial and nuclear phylogenies.

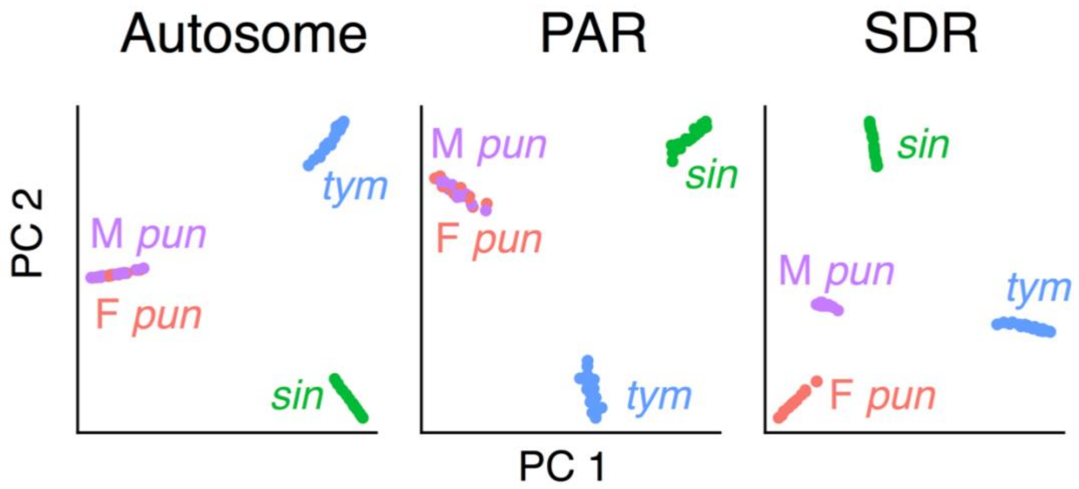

Figure S6: The first two principal components for SNPs from an autosome (Chr I) and the PAR show males and females of *P. pungitius* grouped closely together. But in the SDR, the two sexes separate out, and males are more similar than females to *P. sinensis*.

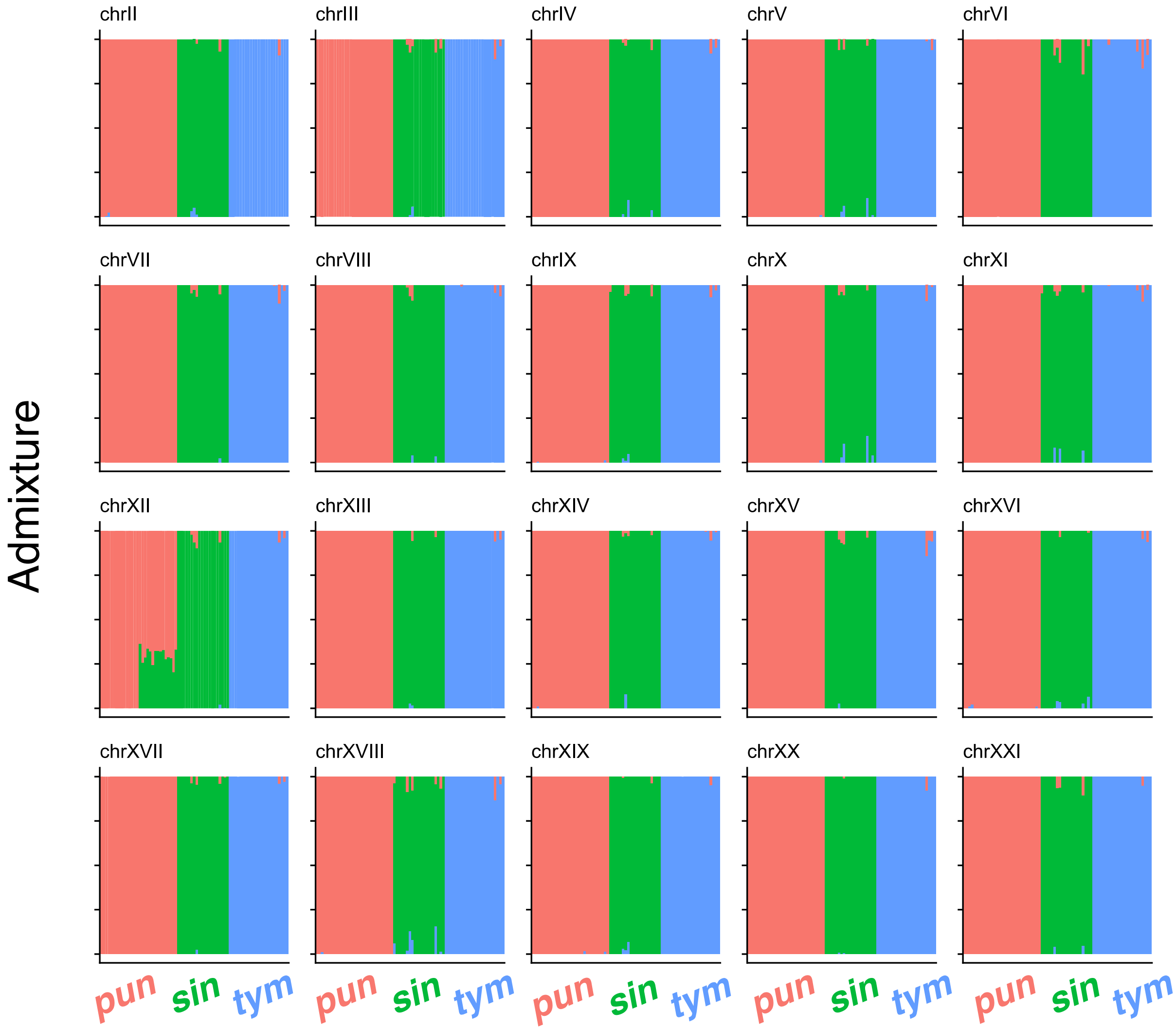

Figure S7: Admixture analyses for the full genome separated by chromosome. SNPs from each chromosome were thinned to retain only a single variant every 10Kb. Admixture analysis was performed assuming three ancestral populations (K=3). With the exception of the sex chromosome (ChrXII), the analysis revealed relatively little admixture between the three species. The plot for Chr I is not included because it is shown in the main text.

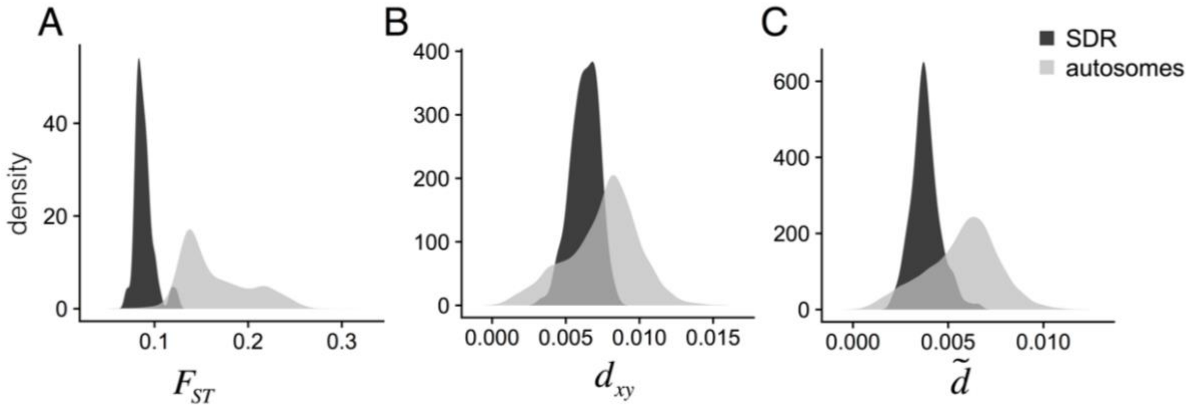

Figure S8: Genetic divergence indicates a recent common ancestor between the *P. pungitius* Y and Chr 12 in *P. sinensis*. Each statistic was calculated for 100 Kb windows taken from the SDR, or the equivalent region of each autosome (all between 4 and 17 Mb; 130 windows per chromosome). (A) Relative genetic divergence (F_{ST}) (B) Average number of pairwise differences per site (d_{xy}). (C) a modified distance measure $\tilde{d}$: $\tilde{d} = d_{xy} - (\pi_x - \pi_y)$; (see methods). The statistic was intended to account for possibility of elevated mutation rates or remaining phasing errors that could inflate estimates of absolute divergence for Y-linked haplotypes compared to the rest of the genome. For each distance measure, the distance for the SDR is less than genomic average, supporting introgression rather than ILS.

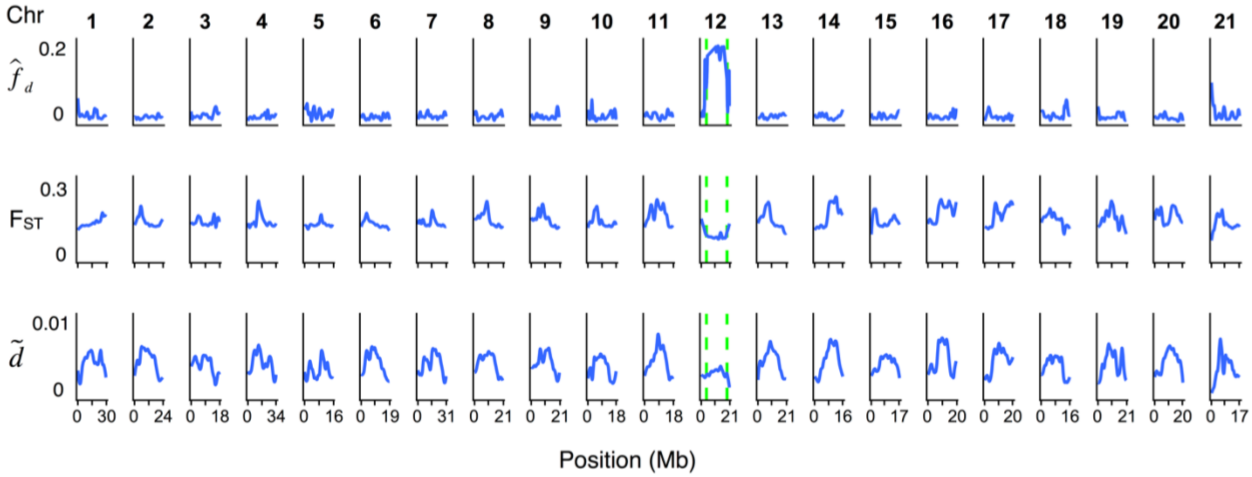

Figure S9: Genomic distributions of statistics supporting introgression. Each column represents the full length of a chromosome with statistics measured for 100 Kb windows. Each row represents a statistic. Row1: $\hat{f}_d$, calculated for shared derived alleles between *P. sinensis* and *P. pungitius* males relative to *P. pungitius* females as indicated in fig. 3 of the main text. Row2: F_{ST} between inferred Y chromosomes and *P. sinensis*. Row3: a modified distance measure $\tilde{d}$: $\tilde{d} = d_{xy} - (\pi_x - \pi_y)$; (see methods), between inferred Y-chromosomes and *P. sinensis*. The vertical dashed lines for *P. pungitius* show the inversion breakpoints at 3.5 and 18.9 Mb identified by Natri et al. (2015).

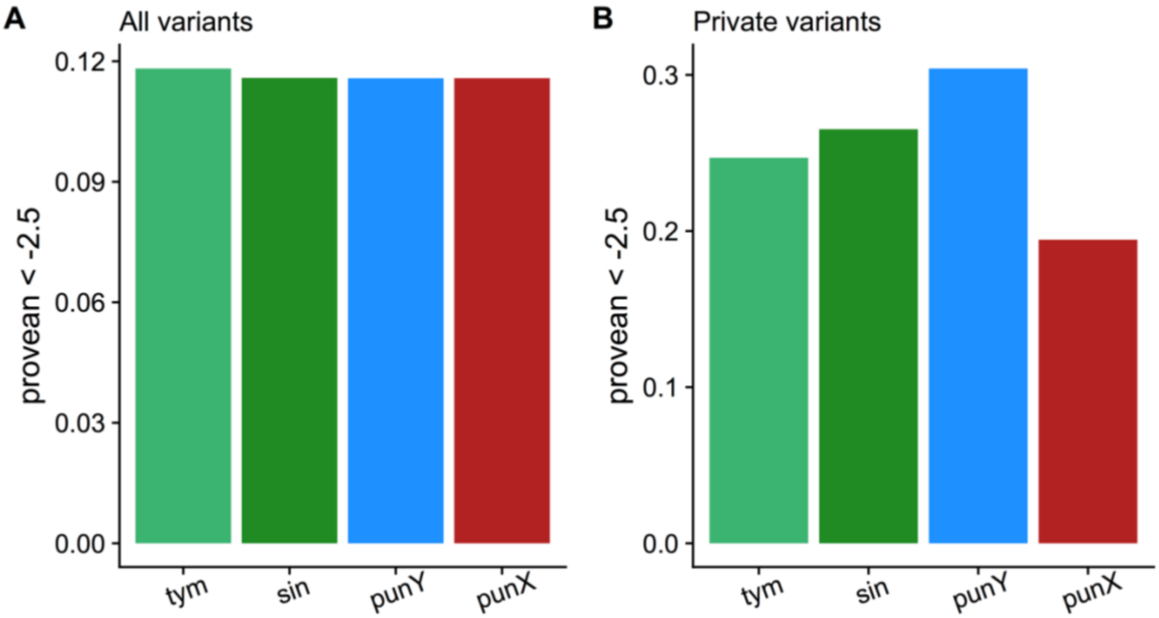

Figure S10: Proportions of amino acid substitutions predicted likely to have deleterious effect based on Provean scores. Amino acid substitutions were predicted to be deleterious based on a Provean score cutoff of -2.5 as in Yoshida et al. (2017). Substitutions were identified relative to the *G. aculeatus* reference. (A) Proportion of all amino acid substations predicted to be deleterious. (B) Proportion of private amino acid substitutions predicted to be deleterious. The difference between the *P. pungitius* Y and *sinensis* was not significant (chi-square test p = 0.42). Although the difference between the *P. pungitius* Y and *P. pungitius* X was significant (chi-square test p < 0.002), the same was true for the other two species. This may indicate that the elevated dN/dS ratio observed for the *P. pungitius* X chromosome (fig. 6D) reflects positive selection more than relaxed purifying selection.

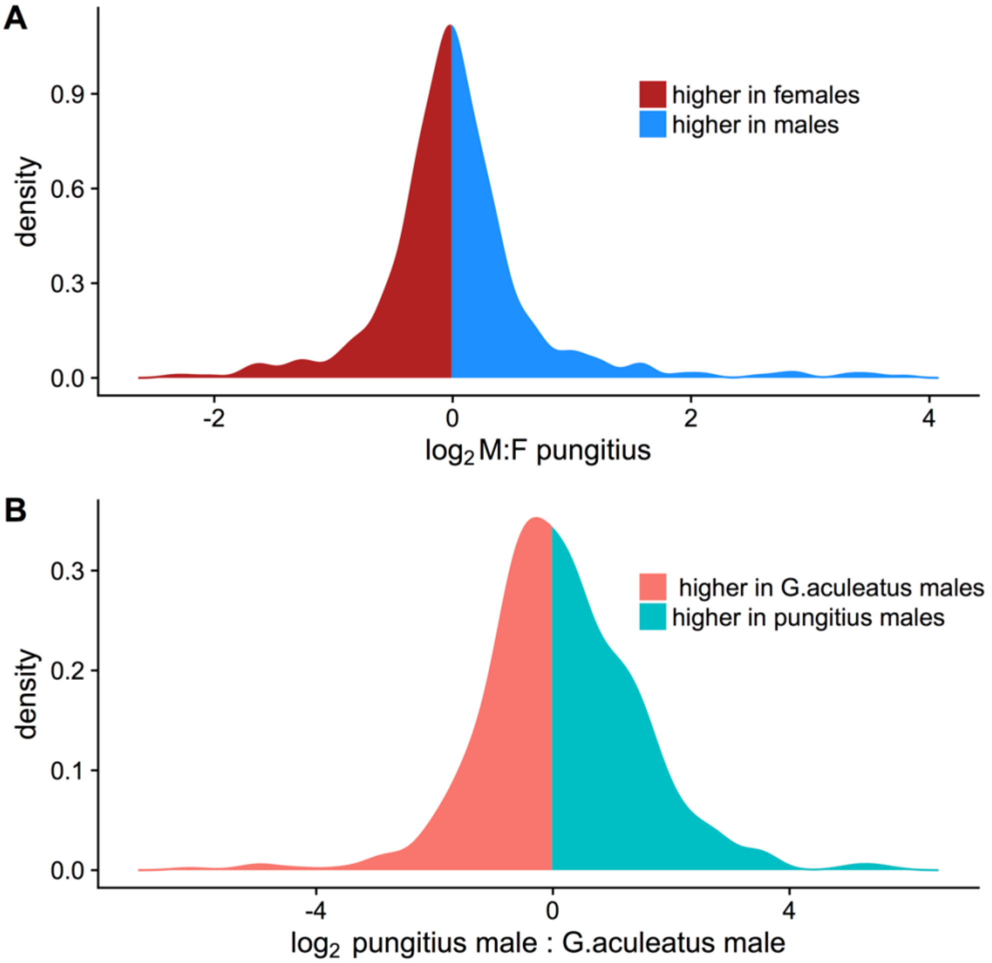

Figure S11: No evidence for preferential loss of Y-linked RNA-seq fold coverage in *P. pungitius*. (A) Distribution of log_2 fold differences for SDR-linked genes between male and female *P. pungitius* (B) Distribution of log_2 fold differences for SDR-linked genes between male *G. aculeatus* and male *P. pungitius*. In both cases, the normal distribution with a mean of roughly zero indicates that transcription has not been preferentially lost from Y-linked gene.

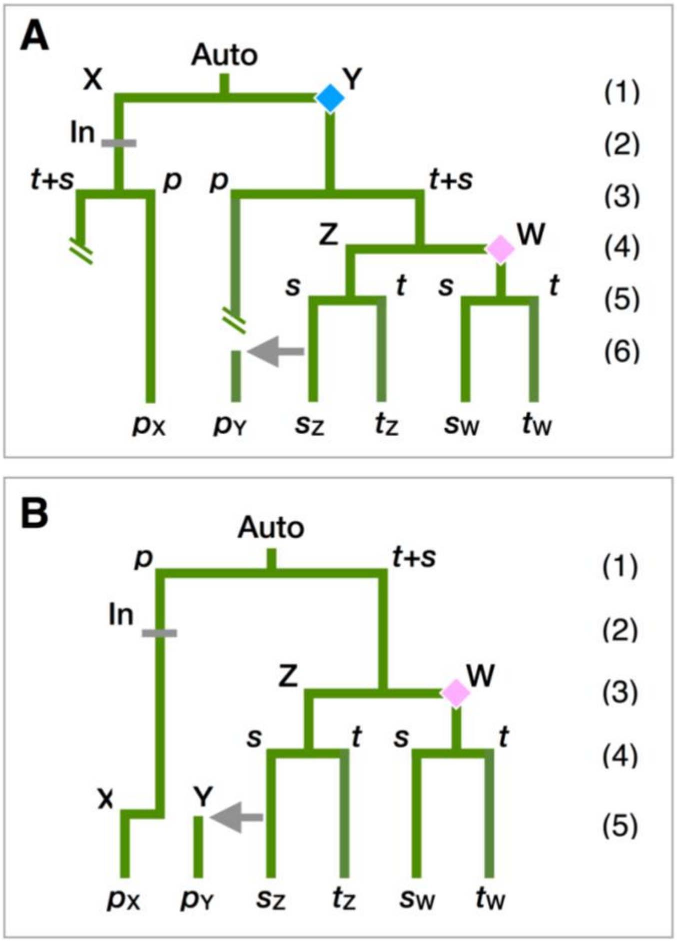

Figure S12: Two hypotheses for the gene tree of Chr 12. (A) Chr 12 carries and XY system in the ancestor of the three species. At time (1), a masculinizing mutation (blue diamond) appears on an autosome, giving rise to the ancestral Y chromosome. At (2), an inversion (In) fixes on the ancestral X chromosome. Following speciation at (3), the ancestor of *P. tymensis* and *P. sinensis* diverges from *P. pungitius*. At (4), a feminizing mutation (pink diamond) occurs on the Y of the *tymensis* + *sinensis* lineage. Establishment of this new W chromosome drives the X chromosomes in that lineage to extinction, while the former Y chromosomes without the feminizing mutation now function as Z chromosomes. At (5), *tymensis* and *sinensis* speciate. Finally, at (6), a Z chromosome introgresses from *sinensis* into *pungitius* (arrow), where it replaces the ancestral Y chromosome. The tips of the tree are labeled by an abbreviation for the species, with a subscript for the type of sex chromosome they now function as. (B) Chr 12 is an autosome in the ancestor of the three species. At time (1), speciation occurs and the *pungitius* lineage diverges from the ancestor of *tymensis* and *sinensis*. At (2), and inversion fixes in the *pungitius* lineage. At (3), a dominant feminizing mutation (pink diamond) appears and gives rise to a W chromosome. Homologous chromosomes without the mutation now function as Z chromosomes. At (4), *sinensis* and *tymensis* speciate. Last, at (5), a Z chromosome from *sinensis* introgresses into *pungitius* (arrow), where it acts as a Y chromosome. The resident copies of Chr 12 in *pungitius* now function as X chromosomes.

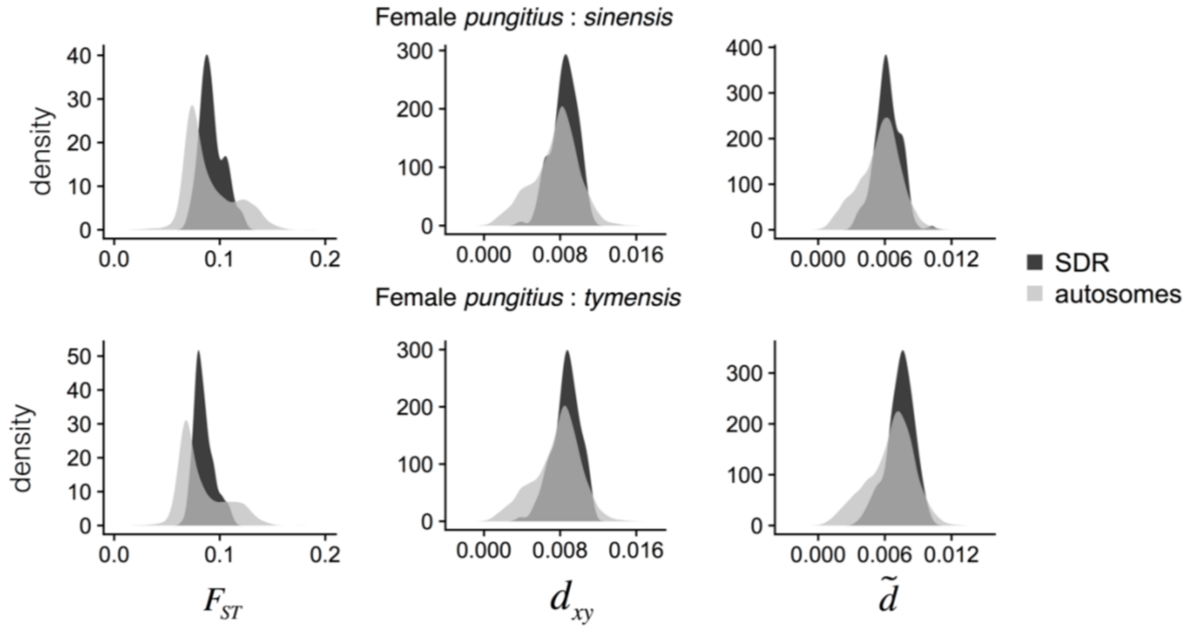

Figure S13: Divergence between *P. pungitius* X is inconsistent with hypothesis that XY system on Chr 12 was ancestral. Density plots illustrate genetic divergence calculated for 100 Kb windows taken from the SDR and equivalent regions of autosomes (all between 4 and 17 Mb; 130 windows per chromosome). If an XY system on Chr 12 was the ancestral state for all nine-spine sticklebacks (Figure S12A), X-linked sequences from *P. pungitius* would tend to be more diverged from *sinensis* and *tymensis* than the genomic average, because they would share a less recent common ancestor. This does not appear to be the case, with distributions for the SDR largely overlapping those for the rest of the genome. Based on these results, we favor the evolutionary history in which the XY system arose within *P. pungitius* (Figure 5B) over the alternative in which XY on Chr 12 was the ancestral state (Figure S12).
